# Present-Day Problems

**Published:** 1888-12-08

**Authors:** 


### The Doctor's Pulpit.

(Continued from page 134.)

### PRESENT-DAY PROBLEMS.

### "Wasters."

In many manufacturing businesses the finished product is subjected to a searching inspection, and is appraised and labelled according to the standard of its value. Every article which does not reach a certain level of excellence is rejected as "inferior." There are degrees of inferiority. Some articles, though inferior, are still of worth. But there is a level below which the finished product is of no value at all. It is then, in business parlance, a "waster." Now, it is obvious that in any business the end to be aimed at is to make the largest possible number of articles such that they shall be worthy to be labelled "excellent." It is equally clear that the fewer the articles which deserve this appellation, and the more which are "inferior," the less successful will the business be as a paying concern. Still as certain is it that if the "excellent" articles are few, the "inferior" more, and the "wasters" most numerous of all in any particular business, that business is a bad one. Bankruptcy is already in sight. That termination is imminent, and the gravity of the catastrophe will be strictly proportioned to the magnitude of the business and the importance of the interests concerned.

This illustration is apt for our purpose. Let a man ask himself what business, in which he is personally concerned, is the gravest and most important of all? He will probably think "his own." But if he will consider it for an hour he will say, if he be a person of any intellectual capacity at all, "the general business of human life around us; the business of the State." Whatever view, however, he may take as an individual, he will at least admit this, that from a public point of view no single private business can be so important to the public at large as the general work and life of the whole community. Human life in communities may be very properly compared with a huge manufactory. The finished products are men and women. It is as certain as certain can be that the laws which govern the prosperity of an ordinary manufacturing business are in full operation in the human community. If in any given social system the finished products are largely of the type which a competent and impartial judge of the human article would label "excellent," that social system will be prosperous and successful. "If the "inferior" specimens of the finished product be disproportionately numerous, there will infallibly be a diminution in the success of the business. But if the finished products yield only a few "excellent" specimens, a large number of inferior, and most of all of the "waster" class, then no providence, human or divine, will save that social system, that organised state, from destruction and ruin.

If this be true, it must always be one of the urgent question of the hour, What proportion of finished human products are "excellent," what proportion "inferior," and what proportion "wasters"? In pursuing an inquiry of this kind it is necessary to agree about a standard of "excellence." The standard must not be too high or too low, but such as will commend itself to the good sense of the reasonable man. Some such standard as this will probably be generally accepted: All men and women who have reached, or are likely to reach, old age in fairly good health, and all men and women who thus far have met, unaided, all their legitimate responsibilities, and who are likely to do so to the end of life—all of this class will be labelled both by the statesman and the physiologist "Excellent." Thoughtless persons may consider the standard too high; but it is not so at all. We surely ought to expect every healthily-born child, who survives the chances and accidents of the child period, to reach old age; and it cannot be considered too great a demand to make upon a man of average health and strength that he shall provide for his own wants. Nature, at any rate, insists that he shall provide for them, for she lays up no stores for the incapable or the helpless, any more than she does for the idle. She knows nothing of poor-laws and casual wards.

But if that be an admittedly just standard of excellence from the statesman's and the physician's standpoint, what we want to know next is: What proportion of the children who are born into the world reach old age, and what proportion provide for all their own wants during the whole of life? A further inquiry should be: What proportion of children who are born into the world fail to approach old age, and what proportion never succeed in providing for their own wants at all? There are no figures available which will furnish an exact answer to these questions; but there are certain well-known facts which indicate with sufficient clearness—at least, an approximate answer. To the philosophic and practical mind, facts are facts, and great facts are great facts, whether they can be set forth in exact measures of feet and inches or not. Here, for example, is one fact of the most striking significance. It is said that one-fourth of the population of London seek gratuitous aid from hospitals in time of sickness. If that be true—and there is no reason for doubting it—we are at once confronted with a quarter of the whole population of London who do not provide fully for their own wants, who are therefore to be classed among the "inferior" specimens of the human finished product. But this by no means exhausts the "inferior" list. No pauper attends the general hospitals. Indoor and outdoor paupers are provided with medical relief by the poor-law. All these must be added to the quarter of the population before specified, only that most of them must be classed as "wasters," and not merely as "inferior." With these, again, are to be reckoned, for our present purpose, all, or almost all, the thieves, who probably as a class seek hardly any medical relief at all. A further addition must be made of the vast numbers of children in poor neighbourhoods, who die because they have no power of survival. All these massed together will probably amount to not less than half the population. Half the population born into the world of London are of the "inferior" type, and a large proportion of these may properly be described as "wasters." The proportions are perhaps somewhat different for the whole country, but the difference is not probably very great.

Will anyone say that these are not significant facts? They are not significant to the fool. Nothing is so significant to a horse as a good clover field, or to a tiger as a fine fat calf. But is man, or is he not, above the intellectual level of the animals whose sole concern is the satisfaction of their immediate and pressing wants? Is he, or is he not, a being who "looks before and after"? From that lowest of all standpoints, personal self-interest, it is surely of some moment that half the population require money help from the other half. When A is reaping the cornfield which his own labour and capital alone have brought to perfection, is it nothing that B, who has not contributed one spade-stroke to the business, shall be looking over the hedge and waiting for his share of the crop? From the point of view of national self-preservation, it is of some significance what position we hold as regards other nations. If our "excellent" finished human articles be fewer in proportion than theirs, and our "inferiors" and "wasters" be more, one result, and one only, is possible as time advances. We must go down, and as certainly they must go up. Many other reasons conspire with these to urge upon all those who have eyes and consciences to use them both; to look deeply and clearly into the facts which lie around them; and then to proclaim boldly and without fear of any man's face that which they see and know to be the truth.

(*To be continued.*)

